# Analysis of Initial Cell Spreading Using Mechanistic Contact Formulations for a Deformable Cell Model

**DOI:** 10.1371/journal.pcbi.1003267

**Published:** 2013-10-17

**Authors:** Tim Odenthal, Bart Smeets, Paul Van Liedekerke, Engelbert Tijskens, Hans Van Oosterwyck, Herman Ramon

**Affiliations:** 1 MeBioS, KU Leuven, Leuven, Belgium; 2 INRIA, Domaine Rocquencourt, France; 3 Biomechanics Section, KU Leuven, Leuven, Belgium; University of California San Diego, United States of America

## Abstract

Adhesion governs to a large extent the mechanical interaction between a cell and its microenvironment. As initial cell spreading is purely adhesion driven, understanding this phenomenon leads to profound insight in both cell adhesion and cell-substrate interaction. It has been found that across a wide variety of cell types, initial spreading behavior universally follows the same power laws. The simplest cell type providing this scaling of the radius of the spreading area with time are modified red blood cells (RBCs), whose elastic responses are well characterized. Using a mechanistic description of the contact interaction between a cell and its substrate in combination with a deformable RBC model, we are now able to investigate in detail the mechanisms behind this universal power law. The presented model suggests that the initial slope of the spreading curve with time results from a purely geometrical effect facilitated mainly by dissipation upon contact. Later on, the spreading rate decreases due to increasing tension and dissipation in the cell's cortex as the cell spreads more and more. To reproduce this observed initial spreading, no irreversible deformations are required. Since the model created in this effort is extensible to more complex cell types and can cope with arbitrarily shaped, smooth mechanical microenvironments of the cells, it can be useful for a wide range of investigations where forces at the cell boundary play a decisive role.

## Author Summary

How cells spread on a newly encountered surface is an important issue, since it hints at how cells interact physically with the specific material in general. It has been shown before that many cell types have very similar early spreading behavior. This observation has been linked to the mechanical nature of the phenomenon, during which a cell cannot yet react by changing its structure and behavior. Understanding in detail how this passive spreading occurs, and what clues a cell may later respond to is the goal of this work. At the same time, the model we develop here should be very valuable for more complex situations of interacting cells, since it is able to reproduce the purely mechanical response in detail. We find that spreading is limited mainly by energy dissipation upon contact and later dissipation in the cell's cortex and that no irreversible deformation occurs during the spreading of red blood cells on an adhesive surface.

## Introduction

The dynamics of initial cell spreading – that is during the first few minutes – are governed by energy release through binding events of cell surface molecules, rather than by active cellular processes such as e.g. tension generated by stress fibers. These molecular binding events dominate the total adhesion energy of the cell. This adhesion creates a pulling effect that in turn generates strong local forces which result in deformations of the actin cortex. The dynamics of initial cell spreading (the increase of radius of the contact area with time 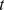) universally correspond to an early (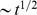), and a later (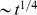) power law behavior [1]. It is only at an advanced stage when the cell is already moderately spread out that active pulling of actin stress fibers on focal adhesion complexes will reinforce cell spreading, depending on the cell type in question, see e.g. [2].

The viscoelastic behavior of the cell boundary is determined not so much by the cell membrane itself but by the intracellular cytoskeleton, or, in the case of red blood cells (RBCs), a network of spectrin filaments directly underlying the membrane [3], [4].

A model that can be used for describing cellular mechanics should be able to accurately describe the mechanical interactions that take place at the cell boundary, i.e. the contact interface with its substrate, the extracellular matrix or surrounding cells. Lattice-free, particle-based methods can describe the interaction forces and the resulting movement and deformation of particles in a natural way. At a point of contact between two particles, contact forces are calculated explicitly based on an appropriate contact force model. From these forces, movement of the particles is calculated by integrating the equation of motion. In the simplest approach, particles are assumed to be spherical. In that case, contact forces can be directly calculated from the sphere-sphere overlap distance 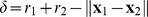 (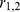 are the radii of the spheres and 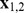 the spacial coordinates of their centers). Calculating contact forces for non-spherical shapes is more challenging: approximations have to be made for the contact force model and it is not trivial to calculate a meaningful overlap distance for all cases. Arbitrary shapes have been modeled by using combinations of connected overlapping spheres [5] or by using polyhedra or poly-arcs, and calculating a contact force proportional to the overlapping volume of the shapes [6], [7]. Besides, the surface of an arbitrary shape can be approximated by sampling points [8]. For each sampling point, a contact force can be calculated based on the indentation in the surface of another object. Disadvantages of using sampling points include that it is hard to directly compare it to a physical contact model such as the Hertz model for spheres, that they generally do not allow to reach complete force equilibrium, and that the precision of the approximation of the contact depends crucially on the local density of nodes, so that the contact parameters need to be re-scaled for different node densities [8].

We present a novel computational framework for describing the mechanical behavior of cells with an emphasis on the interaction between the cell and its environment. Although we only apply this model to cell spreading on a flat surface, the current implementation already allows for more complex settings of interaction with arbitrarily shaped smooth bodies, and cell-cell interaction.

The main novelty of the method developed in this work lies in the fact that we calculate contact between a triangulated surface with “rounded” triangles reflecting the local curvature of a cell and its microenvironment by applying Maugis-Dugdale theory (see section “Maugis-Dugdale theory”) to all contacting triangles. To apply this adhesive contact model for the triangulated surfaces in our models, we build on the following six ideas (see section “Contact mechanics of a triangulated surface”):

1. The triangulated surface can locally be approximated by spheres, i.e. a specific curvature is assigned to each triangle, see section “Local curvature of the 3D shape”.
2. All contact forces are normal to the intersection plane, which is defined by (encompassing) sphere-sphere or sphere-plane intersection. An in-depth discussion is provided by the supplementary Text S1: “Resolution of contact and contact point calculation”.
3. For the approximation of a spherical surface, the sum of all contact forces on the individual triangles must be equal to the appropriate continuum-mechanics force response and the contact parameters should not depend on the chosen mesh. For details on this, we refer the reader to the supplementary Text S2: “Bouncing ball simulation and mesh-independence of contact force”.
4. To integrate the contact force on each single triangle, quadrature rules can be used to calculate approximate pressures in specific points of the triangle. The details of this are discussed in section “Integrating the force on a triangle from the pressure distribution”.
5. Having thus calculated the force on each triangle, it must be distributed to the nodes of the triangulation. This is done such that total force and moments of the pressure contributions on that triangle are conserved. Details are to be found in section “Distribution of force to the nodes of the triangulation”.
6. Finally, an over-damped equation of motion (comparable to [9]) is solved for the nodes of the triangulation, see section “Equation of motion”.

This novel contact model is combined with a new implementation derived from existing mechanical models for red blood cells, mainly from Fedosov et al. [3], [10]. That model has been previously computed using a dissipative particle dynamics (DPD) solver, a different meso-scale simulation method. The mechanical model of the cortex of the RBC includes finitely extensible nonlinear elastic (FENE) connections and viscous dissipation between the nodes of the triangulation, volume conservation and surface area conservation, as well as bending resistance – see section “Elastic model of the cortex”.

**Figure 1:**
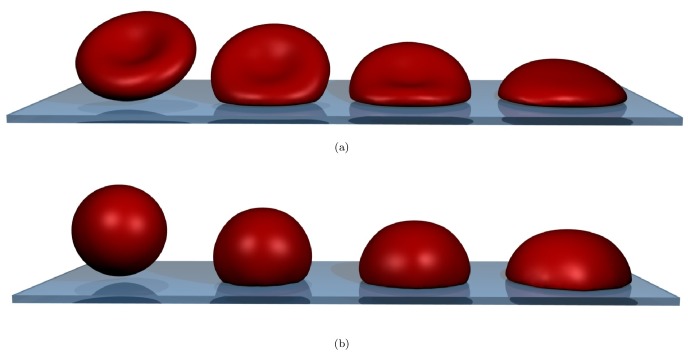
Simulated cell spreading of the red blood cell at three different time-points. (**a**) binconcave RBC spreading. (**b**) “sphered” RBC spreading. From left to right: no contact at 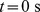, early contact at 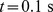, approximately the cross-over between the two regimes at 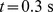 and the fully spread cell at 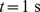. The biconcave RBC has approximately 40% less volume than the osmotically swollen spherical red blood cell. For movies corresponding to these snapshots, see supplementary Videos S1 and S2.

Finally, we apply this newly developed method to an in-depth computational investigation of RBC spreading (see Figure 1 and supplementary Videos S1 and S2) as reported by both Hategan et al. [11] and Cuvelier et al. [1] in order to unravel the governing mechanisms.

## Results

To show the validity of the model assumptions concerning cortex mechanics, we first compare simulated red blood cell stretching to experiments reported in the literature [12]. A combination of FENE potentials with a power law for area incompressibility was used to model the elastic properties of the RBC cortex (see section “Elastic Model of the cortex”).

### Validation of the RBC cortex model

#### RBC stretching experiments

Using the deformable cell model, we perform cell stretching simulations in order to validate the elastic constants of the RBC with respect to optical tweezer measurements, in which a red blood cell is attached to two beads on opposite sides. In the experiment, the beads are pulled apart with a set force, and the deformation of the RBC is measured [12].

To simulate the RBC behavior, we pull on the outermost 5% of the nodes with the same force, and wait until the system is equilibrated. The same parameters as used by [4] in their DPD model yielded comparable results for the presented model – see table 1.

**Table 1:** Parameters used for the RBC-spreading model.

Parameter	Symbol	Value	Units	estimated from:
Timestep*	Δ*t*	6⋅10^−6^	s	trial runs
simulation time	*T_end_*	1.2	s	[1]
conjugate gradient precision	*e_max_*	10⋅10^−15^	N	trial runs
cell radius	*r*	3.25⋅10^−6^	m	surface area RBC [11]
medium viscosity	*η*	0.8⋅10^−3^	Pa⋅s	Blood plasma at 37°C
Young's modulus cortex	*E*	800⋅10^3^	Pa	trial runs
Poisson's ratio	*ν*	0.4	-	[9]
tangential friction coef.*	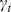	6⋅10^9^	N⋅s/m^3^	“fitted”, [40],[43]
normal friction coef.*	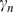	8⋅10^9^	N⋅s/m^3^	“fitted”, [40],[43]
adhesion constant*	*W*	1⋅10^−3^	J/m^2^	[1]
effective adhesive range	*h_0_*	20⋅10^−9^	m	interpolated from [21]
FENE constant (stretch)	*k_s_*	3.2⋅10^−6^	J	[3], [17]
maximal FENE stretch	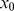	2.05	[-]	[3]
cortex bending constant	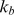	240⋅10^−21^	Nm	[3], [17]
cortex damping	*c*	1.5⋅10^−6^	Pa⋅s	relaxation exp.
local area constraint	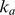	6⋅10^3^	N/m^2^	[3]
global area constraint	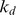	6⋅10^3^	N/m^2^	[3]
volume constraint	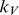	10⋅10^3^	N/m^3^	trial runs conserving *V_0_*

Parameters used for the RBC-spreading model matching data from Hategan et al. [11].

* : Values matching data from Cuvelier et al. [1]: 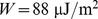 (as reported in [1]), 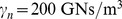, 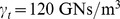, 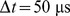.

**Figure 2:**
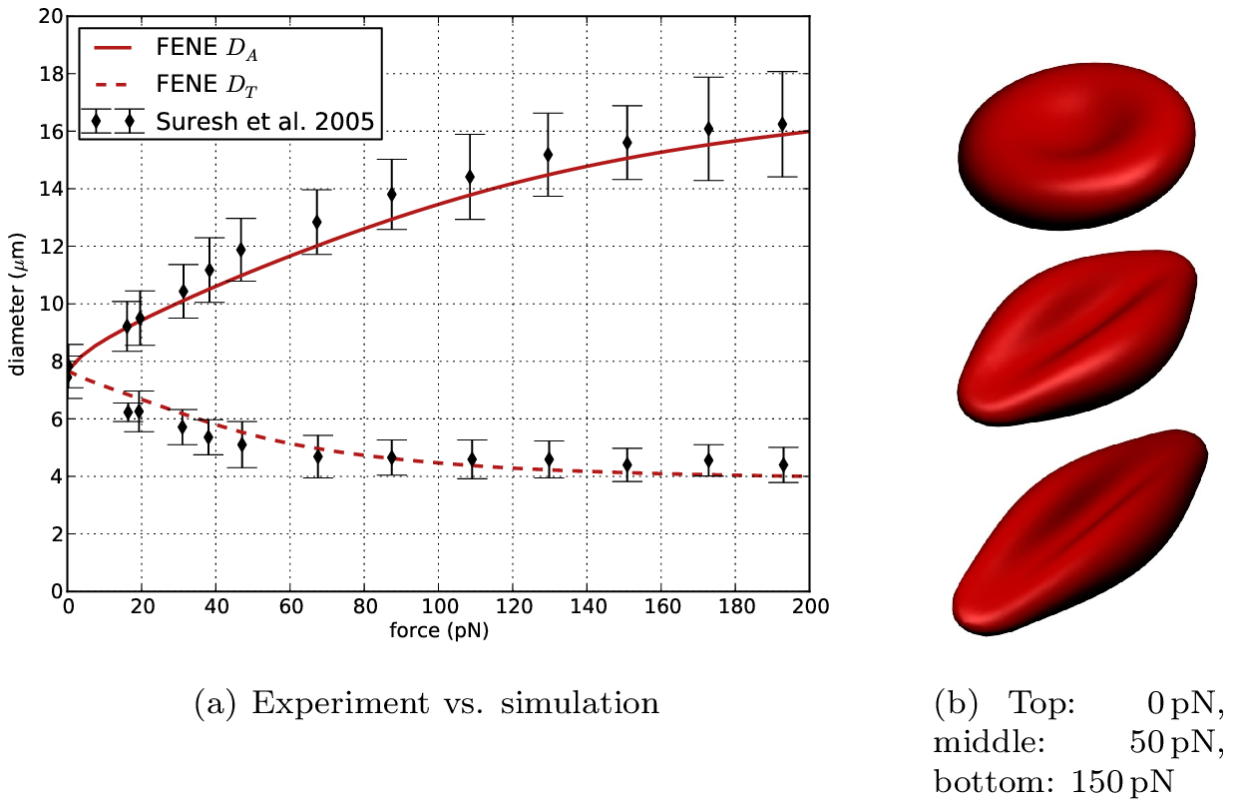
Results of cell stretching. (**a**) shows the change of axial diameter 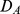 and transversal diameter 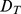 in function of the stretching force, compared to experimental data from Suresh et al. [12]. (**b**) visualizes red blood cells for different stretching forces.

Figure 2(b) gives a visualization of the stretched RBC for stretching forces of 0, 50 and 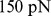. In Figure 2(a) the change in both axial diameter 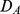 and transversal diameter 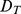 is shown for different cell stretching forces. This curve corresponds well to the computational results presented in the paper of Fedosov et al. [4], who report a maximal axial diameter of 16 µm and a minimal transversal diameter between 4 and 5 µm at a force of 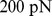, as well as experimental data by Suresh et al. [12].

#### RBC relaxation

**Figure 3:**
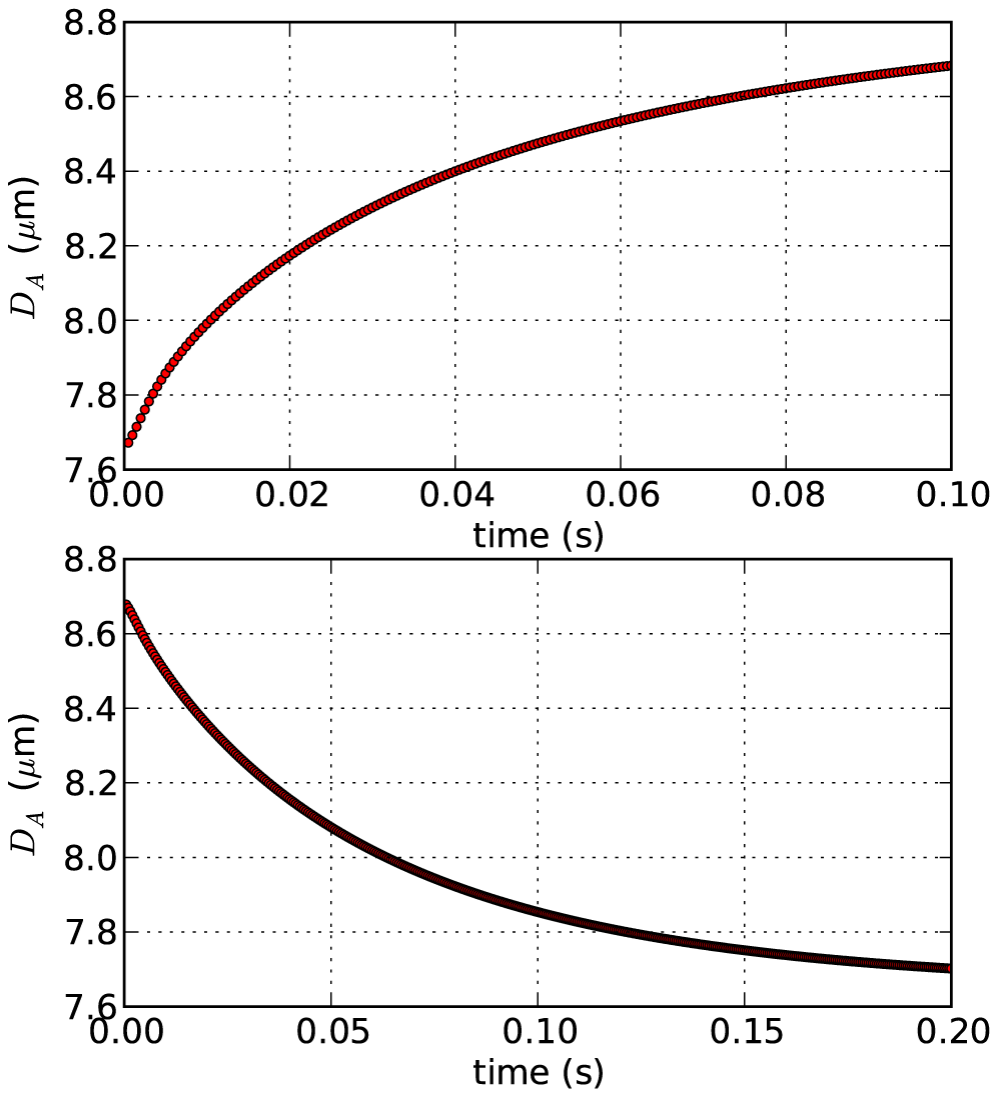
Computational results for cell relaxation. Top: cell stretching dynamics. Bottom: cell relaxation dynamics; cortex damping 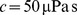.

In order to validate the dissipation constants of the cortex itself (see equation 25), a relaxation simulation was performed. In this *in-silico* experiment, the cell is first stretched with a fixed force until a constant axial diameter 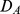 of approximately 8.9 µm is observed. Subsequently, the force is released and the change in 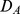 over time is monitored. For a liquid viscosity of blood plasma, we found that the cortex damping coefficient 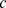 should be chosen in the order of 50 µmPas to match experimentally observed RBC relaxation dynamics (Figure 3). In this case, the computational results are in good agreement with experimentally observed RBC relaxation times in the order of 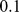
–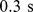 [13].

### Cell spreading experiments

In the experiments reported by Cuvelier et al. [1], biotinylated RBCs were osmotically swollen to become spherical and the change of the radius of the contact area with time was measured for spreading on a streptavidin coated surface. To compare to the spreading dynamics reported in that paper as well as by Hategan et al. [11] (where the cells spread on a polylysine coated surface), we set up simulations of the described model with the parameters as given in table 1.

The red blood cell is modeled with a viscoelastic cortex including bending stiffness and Maugis-Dugdale contact interactions. Most parameters in table 1 are taken directly from the literature as indicated. The effective range of interaction 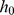 (see equation 8) was estimated at 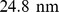 by interpolating from [14] for cells with a radius of ≈3 µm. The cortex Young's modulus used in the Maugis-Dugdale model is the material stiffness of the phospholipid-spectrin complex (the elasticity of the deforming membrane is already taken into account by the FENE potentials). This material stiffness can be assumed to be much higher compared to the whole cell's Young's modulus and is set at a value of 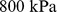. The parameters for the cortex are validated by performing the cell stretching and relaxation experiments explained in the previous section “Validation of the RBC cortex model”.

### Visual and static comparison to data

A view on three stages of the cell spreading for both biconcave and sphered RBCs is presented in Figure 1. Note that the volume of the biconcave RBC is only about 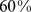 of the volume of the sphered RBC. As a result of that, for the sphered RBC, the final height of the spread-out cell is greater and it has a higher angle of contact compared to the final shape of the initially biconcave RBC.

For this simulation, a triangulation based on a five-fold subdivision of an icosahedron was used – see section “Generating triangulated meshes of cells”. This level of mesh refinement is required to reproduce the final high curvatures at the edge of the contact area when the cell is fully spread out: The triangles at the edge have encompassing spheres with radii of ca. 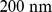, while Hategan et al. [15] report a typical radius of the rim for this situation of 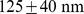, which is of comparable order of magnitude.

The shape of the final spread-out cell is a spherical cap. By fitting a sphere through the top 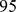
% of the nodes, the effective contact angle [16] can be estimated. For the modeled RBC, we calculate an effective contact angle of 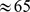, which corresponds reasonably well to the measured effective contact angle of around 60° [15].

### Comparison to dynamic data & influence of parameters

**Figure 4:**
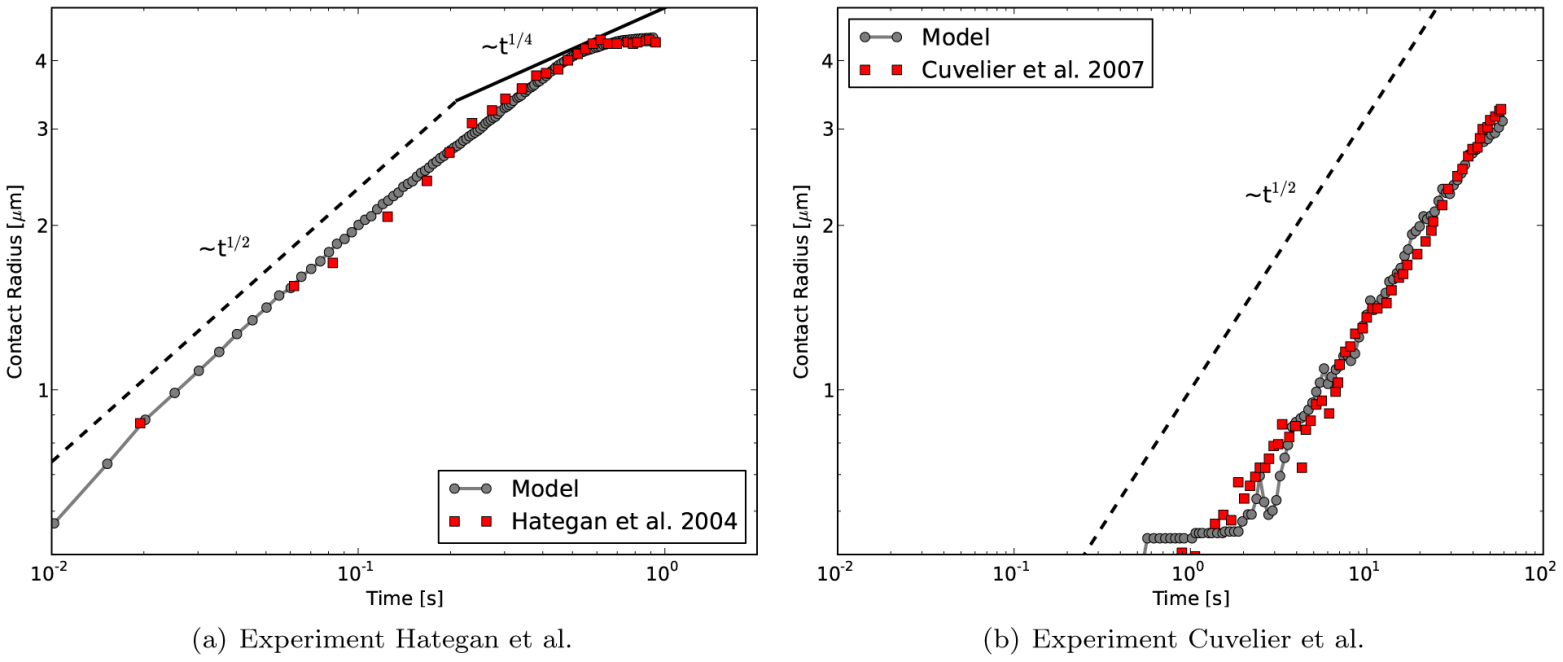
Contact radius vs. time for cell spreading simulations. comparison with experimental data from (**a**) Hategan et al. [11] for adhesion strength of 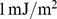 and with data from Cuvelier et al. (**b**) for adhesion strength of 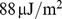 – here, we use a coarser mesh with 642 nodes instead of 2562 nodes since the cell does not spread completely in the given time-frame and therefore does not exhibit the high local curvatures as in the Hategan et al. experiment.

Figure 4 shows the power-law behavior of the sphered RBC spreading in double logarithmic representation. The “contact radius” of the RBC 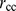 in these and the following figures is calculated from the sum of all the triangles' areas which are in contact 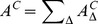 by defining 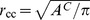. The spreading dynamics of the model match the experimentally observed cell spreading [11] very well.

**Figure 5:**
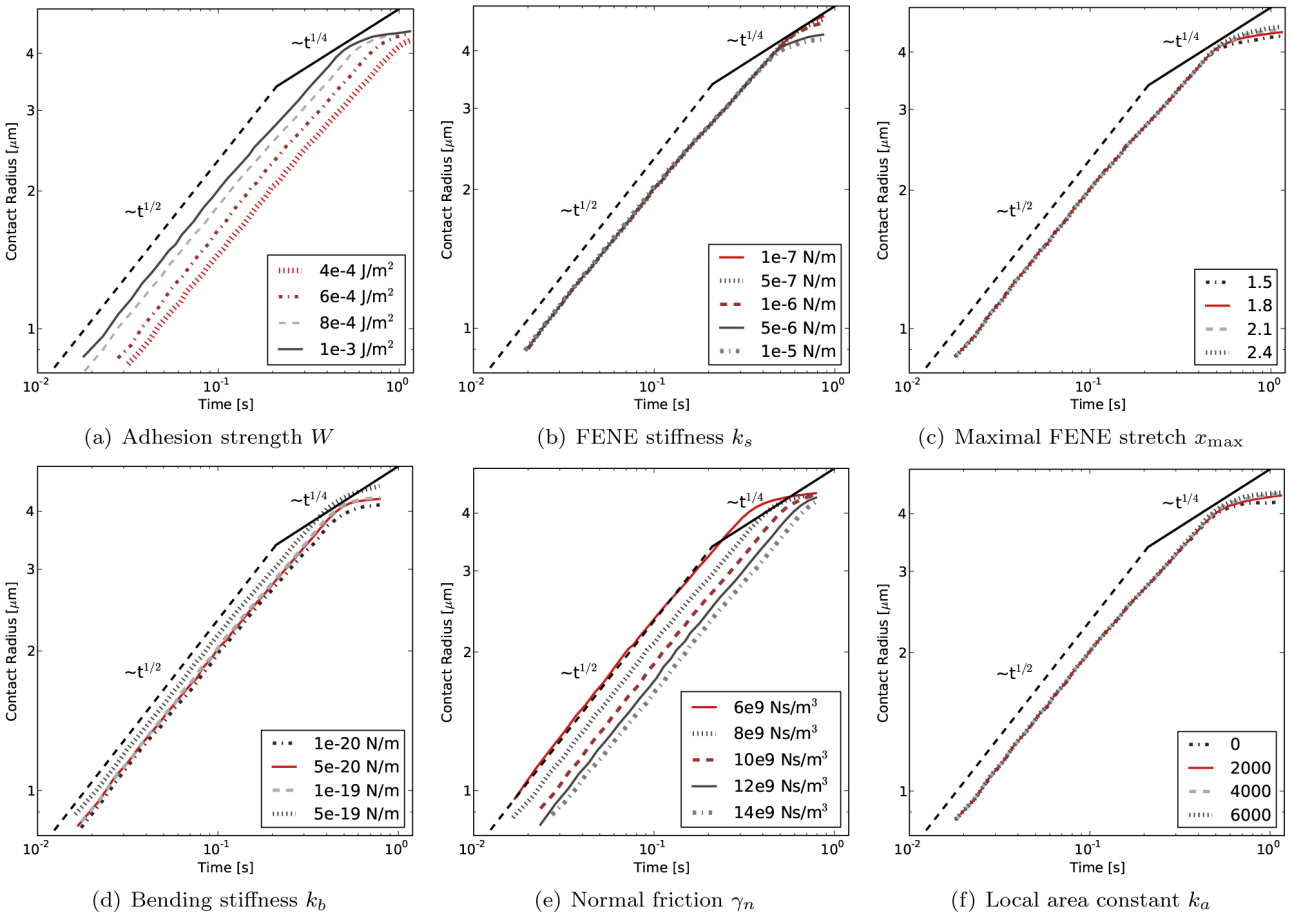
Variation of most influential model parameters. Double-logarithmic plots of cell contact radius 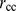 versus time. (**a**) varying cell-substrate adhesion strength 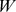 yields both a shift in speed and final contact radius. (**b**) varying the FENE stretching constant 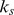 yields different final contact radii, (**c**) varying the FENE max strain 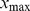 also mostly influences the final contact radii, (**d**) varying bending stiffness 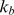 influences both spreading speed and final contact radius, (**e**) varying the normal friction coefficient 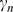 influences spreading speed and (**f**) varying the local area constraint constant 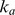 influences the final spreading radius. For a comparison of spreading rates, see supplementary Figure S1.

Figure 5 summarizes the influence of varying one parameter at a time for the most influential parameters of the model starting from the base parameter set reported in table 1. Its first sub-figure (a) shows simulation results of cell spreading for different values of the cell-substrate adhesion strength 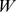. A lower adhesion strength results in a lower final contact radius, but also makes the spreading slower. However, the 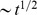 power law behavior as reported by Cuvelier et al. [1] stays well conserved for different adhesion strengths.

The influence of the FENE stretching constant 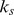 is shown in Figure 5(b). In the range of the RBC FENE constant (in the order of 1 µN/m), the influence of 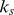 on the spreading dynamics is comparatively small. For larger deviations, higher values of 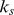 limit the final spreading radius to a lower value, or conversely, lower values allow the cell to spread considerably more.

A FENE connection is also characterized by the maximal stretch 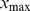 (Figure 5(c)), which expresses the maximal extension of the spring, at which the FENE force diverges (equation 22). The initial spreading dynamics are not affected by the precise value of 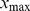, but the final spreading radius is. For higher values of 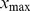, the same tension in the cortex corresponds to a larger extension and therefore a larger radius of the spread out cell.

The effect of the bending stiffness on RBC spreading is shown in Figure 5(d). A higher bending resistance of the cortex speeds up cell spreading, the probable reason being that, through resisting to bending, the cortex keeps the contact angle within the effective range of adhesive interaction close to 180°. This range is of the order of 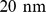 for microscopic biomolecular surfaces [14]. It should be noted that for a theoretical vesicle with bending resistance, the actual contact angle is always 180° [16]. However, for a real RBC, the width of the adhesive spreading front is non-zero and determined by the effective range of interaction 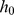. This effective adhesive range is taken into account in Maugis-Dugdale theory (equation 8) and relates the maximal adhesive tension at the edge of contact to the total work of adhesion 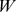.

The normal friction coefficient 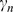 is determined by the energy dissipation when adhesive contact is initiated. The dissipation is caused by snap-in-contact events when adhesion molecules form bonds, and the hysteresis arising from unbinding stochastically again [14]. In Figure 5(e), the effect of changing 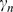 on the RBC spreading dynamics is shown. As could be expected, a lower value of 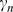 diminishes the energy dissipation due to adhesion and therefore increases the rate of cell spreading. However it does not change the initial 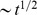 power law behavior of cell spreading.

Finally, in Figure 5(f), the effect of the local area constraint on the spreading dynamics is shown. When the value of 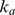 is too low, degenerate triangle shapes can arise with a strongly decreased area. This will result in an underestimation of the final spreading radius. It can be observed that for values of 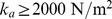, the local area of the triangles is sufficiently well conserved and the predicted spreading dynamics are not affected.

### Evolution of forces acting on the cell

**Figure 6:**
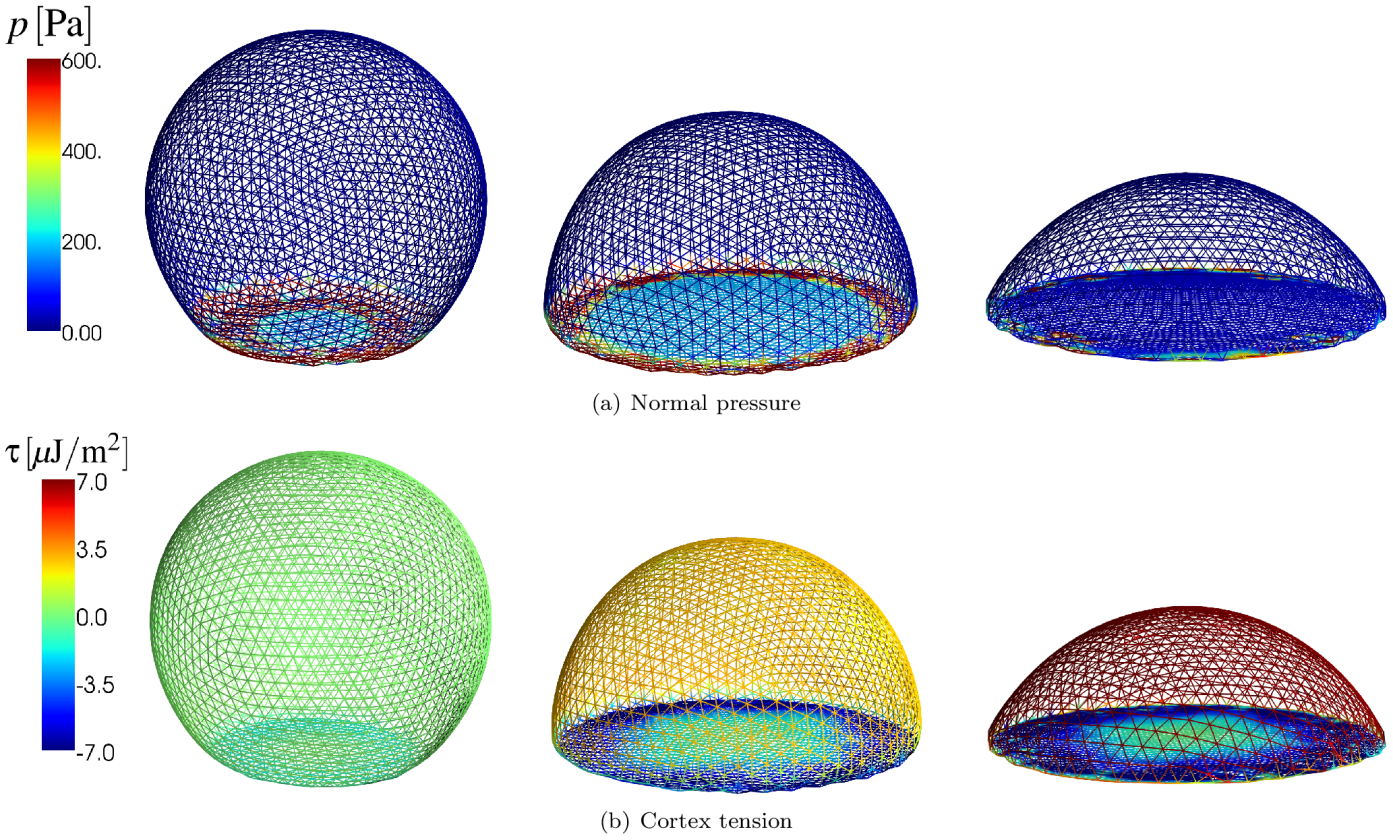
Normal pressure and cortex tension of a spreading RBC. (**a**) Normal pressure (the magnitude of the sum of all conservative forces projected onto the normal in that node) at different time points during cell spreading. left: 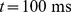, middle: 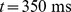, right: 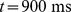. (**b**) Cortex tension (see equation 1) averaged at the nodes during cell spreading at the same time points. In the supplementary Video S2 the sum of all conservative forces acting at each node is indicated by small arrows which are mostly visible for the out-of-plane forces. The distribution of stretch in the cortex is visualized in supplementary Figure S2.

In Figure 6(a), the outward normal pressure on the nodes is visualized for three distinct phases of the cell spreading process for a sphered RBC. The normal pressure is defined here as the magnitude of the sum of all conservative forces (on the left-hand side in the equation of motion, 31) in the nodes projected onto the normal in that node – therefore this normal pressure is dominated by contact forces, where adhesive ones yield a positive (outward) pressure in this case. Figure 6(b) shows the in-plane tension 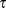 (in 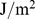) of the cortex (further denoted as cortex tension, and not to be confused by the adhesive tension, given by Maugis-Dugdale theory, see equation 7) at the same time points. This tension is characterized by the FENE force at the inter nodal connections. Positive forces in these connections correspond to tensile stress in the cortex, while negative values are associated with compressive stress:
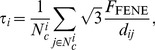
(1)where 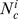 is the number of FENE connections of node 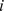 and 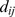 is the inter-nodal distance (see e.g. [17]).

At 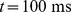 the spreading dynamics correspond to the 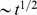 power law regime. At this stage, adhesive forces are strong especially at the edge of contact, but also in the entire rapidly increasing circular contact area. The elastic energy stored in the membrane at this point in time is very low, as the stretch and bending in the membrane is small. As a result, almost all the energy dissipation (see section”Equation of motion”) takes place in the contact area.

At 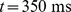, a distinct adhesive edge can be observed, in which the magnitude of forces is much stronger than in the inner circle of the contact area, where the contact potential is already nearly minimal. At the edge, the cortex's bending stiffness provides resistance to the strong adhesive tension. Meanwhile, the upper spherical cap is being stretched while at the plane of contact the membrane – together with the substrate it is adhering to – is under compressive stress. At this stage, energy dissipation takes place not only at the substrate interface, but also in the entire stressed cortex. As a result of this, the spreading slows down to a lower rate than the 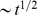 power law regime.

At 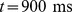, spreading has stopped and the cell has reached equilibrium. The forces at the nodes are zero, and the adhesive tension at the edge of contact is being balanced out by the elastic stress in the RBC membrane/cortex. The cortex in the spherical cap is under strong tensile stress and the stretch in the connections is close to its maximal value 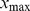. At the substrate interface, compressive stresses have built up even more. For an elastic substrate, these compressive forces will cause radial inwards deformation of the substrate, as has been observed in traction force microscopy measurements [18], [19] – although these experiments concern late cell spreading.

It should be noted that the maximal normal pressure at the nodes – occurring in the first stage of cell spreading – corresponds to a force in the order of 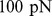, which is in the range of the force applied in the stretching simulations which were used to validate the model parameters of the elastic cortex, see section “RBC stretching experiments”.

## Discussion

### Model performance and limitations

First, with regard to the performance of the newly developed model for a triangulated, deformable cell obeying Maugis-Dugdale contact tractions, we conclude that:

1. We can reproduce the quasi-static cell stretching experiments as analyzed by [3], [4] with nearly identical parameters although the simulation technique used is different (DPD vs. first-order equation of motion inspired by Stokesian dynamics [20]) – see section “RBC Stretching experiments”.
2. The model recapitulates the mechanical behavior of a spreading red blood cell with high precision. From known mechanical parameters it accurately reproduces the cell spreading curves experimentally obtained by [11] and [1].
3. Contact calculations between (rounded) facets of the triangulation show three important advantages over naive node-node based contact calculation schemes:
  - Parameters are physically meaningful, well defined and (in principal) measurable;
  - using these parameters for different mesh refinements yields very similar results (see also supplementary Text S2) for cell spreading, and
  - the desired accuracy is tunable – both by choosing a finer mesh or more quadrature points for higher accuracy, as needed.
4. The dynamics of both experiments (RBC on polylysine-coated glass, biotinylated RBC on streptavidin substrate, [11], [1]) can be matched by only changing the adhesion energy as given by [1] and adjusting the friction constants 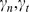 (Table 1). The contact dissipation cannot be expected to be identical for these two situations, since in the first case, the cell is completely spread within a second, whereas in the second case it takes about a minute. Therefore, rates, numbers and nature of binding/unbinding events will be vastly different, giving rise to different dissipation levels (for a more thorough explanation, see e.g. [21], chapter 9.4).
5. The use of a FENE-like potential is important to consistently obtain these spreading dynamics (data not shown). The same behavior cannot be captured by simple linear springs since they would be either too stiff to allow the initial “fast” spreading phase, or too soft to keep the cell from spreading out too much when the adhesion driven spreading stops. The FENE potential captures this initial softness and final stiffness of the spectrin connections very well (see Figure 2). As a result, the predicted spreading dynamics are very robust – no reasonable change of any parameter yielded anything but an initial 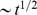 spreading.
6. A five-level subdivision of the icosahedron is required to accurately model the high curvatures occurring when the cell is fully spread out – see section “Visual and static comparison to data”. Using a lower order triangulation yields very similar initial spreading dynamics, but fails to reproduce the final spreading radius of the cell.
7. The model is general enough to allow for simulations in more complex situations – cells interacting with smooth shapes, cells interacting with other cells, etc. It is also well suited for inclusion of cytoskeletal elements (such as the actin network, microtubules, nucleus) in a discrete way.

The modeling technique described in this work has a number of limitations:

- The mesh that is used needs to be refined enough to capture the smallest structures/curvatures that are of interest in the system. This results in comparatively expensive simulations or the additional complication of re-meshing in appropriate regions.
- The linear approximation for the dissipative forces in the equation of motion must be regarded as a first-order approximation of a very complex phenomenon: e.g. [14] notes, that the dissipation upon contact is a time-scale dependent effect, which indicates the limited applicability of the “viscous friction constants” (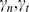). This is the reason why we could not match both observed spreading curves in the experiments by Hategan et al. [11] and Cuvelier et al. [1] with the same values for 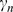 and 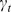. For cell spreading that happens at the same time scale with similar materials involved, we expect the constants to be very similar.
- The current state of the model does not describe the phenomena affecting late cell spreading which are relevant for other cell types. The dynamics of this active spreading are regulated by cellular processes such as actin polymerization, formation of focal adhesion complexes and stress fibers. Models incorporating the biological effects occurring during late cell spreading have been described [22], [23]. However, they cannot directly relate the initial spreading dynamics to material properties such as adhesion strength and contact dissipation.

### Understanding initial cell spreading

Finally, regarding the initial dynamics of cell spreading, we find:

1. The “universal” [1] 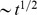 power law behavior of initial cell spreading is found consistently. Moreover, this behavior is very robust to changes in model parameters, because it is caused by geometrical properties of the spreading cell. From the simulations we observe that this first spreading phase is characterized by the absence of tension in the cortical membrane. Since almost no forces are present there, little energy is stored elastically or dissipated in the cortical shell. To understand the 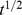 power-law for the radius of contact, we follow the analysis presented by Cuvelier et al. [1]. We conclude that the energy dissipation rate is mainly affected by contact dissipation due to friction. It is therefore proportional to 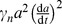, which can be balanced by the adhesive power. This adhesive power (rate of adhesion-energy gain) is proportional to 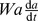, yielding for the trivial integration (ignoring all constants)
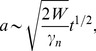
(2)which explains (assuming the given approximations) the characteristic 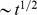 power law dynamics for the contact radius 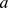. Summarizing, the total energy dissipation per area which is coming into contact with the substrate is constant at this very early stage of cell spreading, yielding the observed dynamics.
2. The first, “fast” slope can only be maintained until the cell's cortex is under tensile stress: In that case, spreading further dissipates more energy – the stretching deformation causes viscous dissipation in the dashpot-like elements, while some is also stored in the (still weak) FENE-like potential. Cuvelier et al. [1] show for several cell types, that in this region a second power law 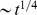 can be found, but it is least pronounced in the experimental RBC data (see Figure 4(a)). From the simulations we observe that there is no clear second power-law regime, but merely a slowing down of the spreading.
3. The final spread-out phase is characterized by a high tensile, in-plane stress in the spectrin-phospholipid cortical shell. This stress is caused by the balance between adhesion forces that occur at the edge of the spread out cell (in the flattened out center, repulsive and adhesive forces balance out and the contact force is very low) and the FENE connections approaching their maximum extension in the upper spherical cap. The adhesive tension at the edge also causes the membrane-substrate interface to be compressed in a radially inward direction. For a substrate that has shear elasticity, the model therefore predicts that the substrate would deform in a radially inward direction. This prediction is in good agreement with experiments using Traction Force Microscopy [18] – although these experiments are more concerned with the late, active cell-spreading state.
4. Most of the energy dissipation during initial cell spreading occurs due to contact dissipation. The simulations indicate that for a red blood cell, no irreversible deformation in the cortical shell is required to reproduce the experimentally observed spreading dynamics. This means that, should we pull back our cell from the substrate, the cell would re-gain its initial shape, as the equilibrium lengths of the FENE connections and the equilibrium angles of the bending connections have not been changed. This is contrary to the simpler, conceptual model proposed by Cuvelier et al. [1], which relies on the dissipative “flow” of the cytoskeleton for energy dissipation.

Although the model as shown is restricted to RBC spreading dynamics, we expect that these conclusions can be generalized to other cell types: the same key mechanical components are present in other systems as well, and despite the fact that other cells' cytoskeletons are more complex and the cells can dissipate energy through “active biological processes”, we expect the initial cell spreading phase to be still characterized by contact dissipation. Eventually, stress in the membrane/cortex will build up as well and through this, the cell will dissipate energy in the entire cortical shell. However, it is possible that this dissipation involves irreversible deformation in the cortex.

## Models

To explain the model developed in this work, Maugis-Dugdale theory is briefly summarized. Building on this theory, an in-depth description of the application of this theory to the contact mechanics of a cell with its mechanical microenvironment is given. Finally, we explain the integration of that model with an existing mechanical model for the cortex of a red blood cell.

### Maugis-Dugdale theory

**Figure 7:**
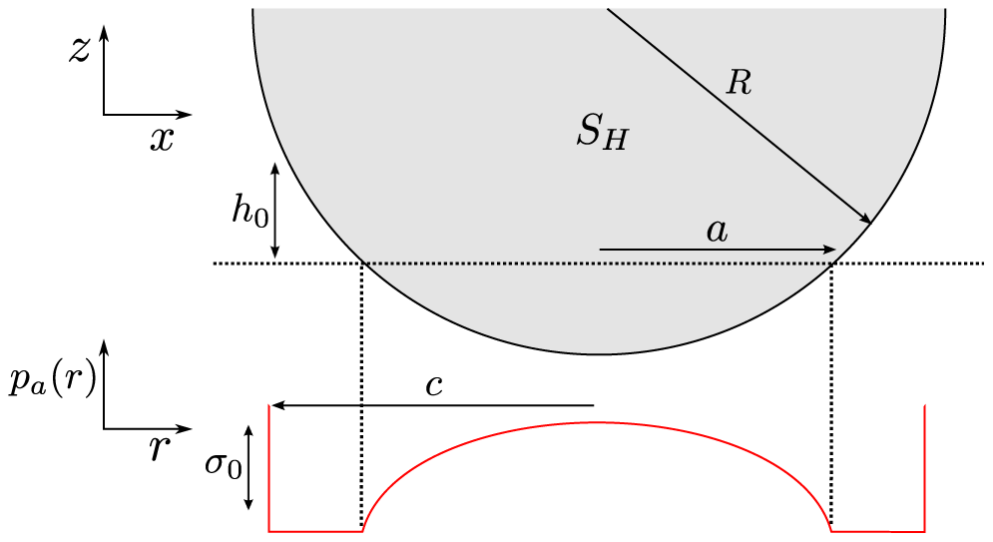
Half-sphere 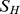 with radius 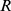 indenting a flat plane and adhesion stress 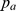 according to the Maugis-Dugdale model.

For two spherical asperities in contact or one asperity in contact with a flat surface (see Figure 7), Maugis-Dugdale (MD) theory can be used to describe the contact mechanics [24]. This theory captures the full range between the Derjaguin-Muller-Toporov (DMT) zone of long reaching adhesive forces and small adhesive deformations to the Johnson-Kendall-Roberts (JKR) limit of short interaction ranges and comparatively large adhesive deformations in the transition parameter. This transition parameter 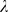 relates to the Tabor coefficient by a factor of 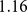 [25].
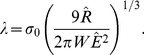
(3)In equation 3, 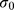 is the maximum adhesive tension (measured in Pa) from a Lennard-Jones potential, 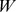 (in J/m^2^) the adhesion energy, 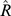 is the reduced radius of the asperities and 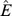 the combined elastic modulus:
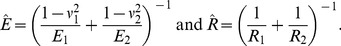
(4)


The (repulsive) Hertz pressure associated with a contact of radius 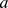 (see Figure 7) is given by
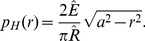
(5)


Assuming a spherical asperity – and therefore a circular contact area – the total Hertz force can be calculated by integrating equation 5 over the complete circular contact area with radius 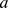, which yields the total Hertz force:
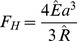
(6)An adhesive stress can be formulated as [24], [26]:
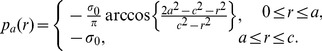
(7)


In the Maugis-Dugdale model, local adhesion tension is assumed to be independent of the overlap until a cut-off distance 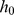. If the asperity is further than 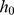 away from the flat surface, the adhesive tension drops to zero. Therefore, 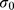 is related to the adhesion energy 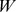 as:
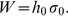
(8)

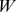
 is the total work of adhesion, i.e. the work required to move the asperity away from the surface and out of contact. To pull a small area 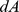 out of contact, the required work 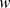 is:
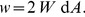
(9)


The total (global) adhesive force is the integral over the adhesive zone with radius 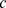 (see Figure 7), which according to [26] becomes:
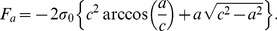
(10)The force in equation 10 is dependent on 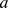. As equation 10 expresses the global adhesive force of the complete asperity, it is not a constant force, but through 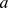 dependent on the indentation. To calculate the adhesive radius 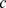 from the actual geometrical contact area with radius 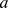, the height at the edge of the adhesive zone 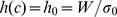 can be used. Substituting both repulsive and adhesive pressures at 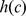 (see equation 5 and 7) this yields [25]:
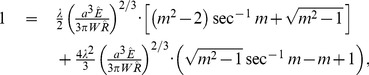
(11)where 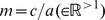. In general, to calculate both 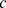 and 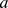 from a given state of the contact, one needs to solve this equation simultaneously with the equation for the net contact force [25]:
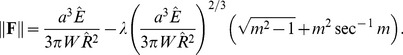
(12)


A very well validated contact model for soft, adhesive bodies like cells, the JKR theory [27]–[29], is a limiting case of Maugis-Dugdale theory for negligible cutoff-distance for the adhesive interaction 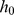 (or 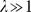) . It has therefore a parameter less than MD theory. The adhesive pressure according to JKR (compare to equation 7) is
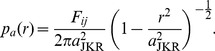
(13)Note that this pressure diverges at 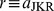.

Summarizing the Maugis-Dugdale theory for an adhesive contact, one considers three distinct zones:

- The Hertz-zone with contact radius 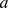, in which Hertz' theory determines the repulsive pressure. Apart from that, there is also an adhesive tension present in this contact zone.
- A purely adhesive zone with width 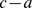, in which no actual contact is formed but a constant adhesive tension is present. The adhesive force in this zone is determined by comparatively long-range interactions.
- At the edge of that adhesive zone, no interactions take place anymore, and contact pressures and tensions vanish.

### Generating triangulated meshes of cells

**Figure 8:**
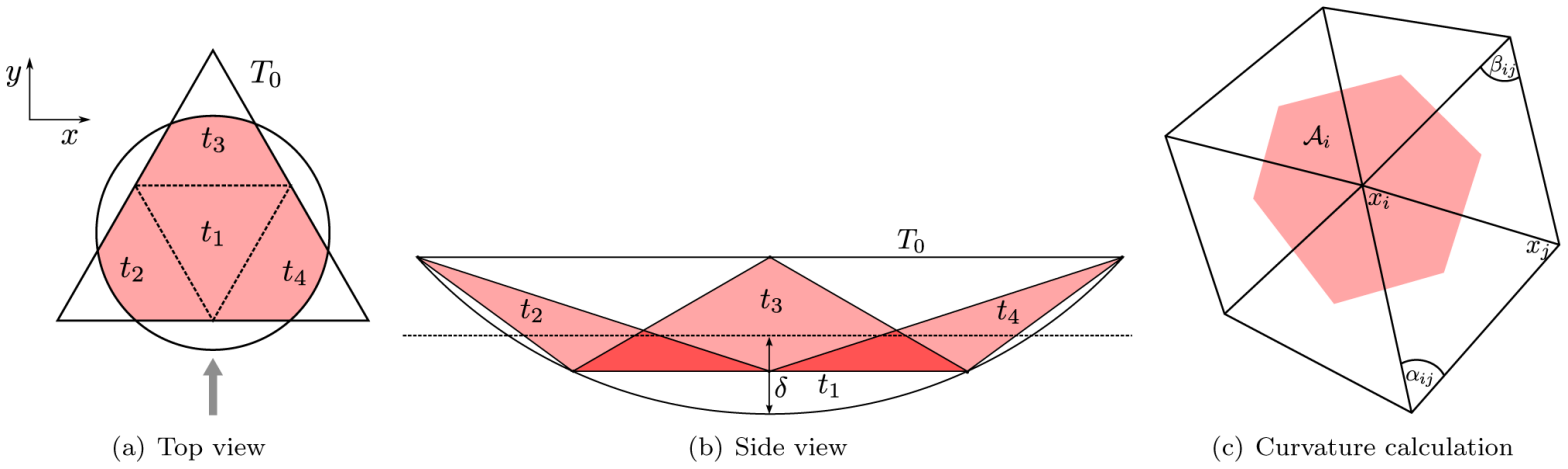
Geometrical properties of triangulations with local curvatures. The top view (**a**) indicates the line of sight of the side view (**b**). (**c**) The contact between the cell boundary and external structures is calculated from encompassing spheres over the triangles with an inverse curvature that matches the local surface curvature. The drawing provides the geometrical definition of the Voronoi region area 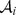, angles 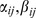 and points 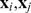 as used in equation 14.

The meshes used in this work are derived from spherical shapes by subdividing an icosahedron and projecting the nodes on a sphere [30]. In a subdivision, each triangle gets split into four triangles as is illustrated in Figure 8. Here it is shown how one triangle with an encompassing sphere matching the local curvature of the cell, is split into four triangles. Since the local curvature is kept, the new triangle nodes are all located on the surface of the same encompassing sphere. Every subdivision of an icosahedron has only twelve nodes with a five-fold connectivity and slightly longer distances to their neighbors; otherwise, the mesh is perfectly regular with six-fold connectivity and is ideal for curvature calculations (see section “Local curvature of the 3D shape”) as reported by [31].

The bi-concave shape of an RBC can be obtained by reducing the volume of the sphere to approximately 60% , and letting a system of linear springs with appropriately chosen parameters relax again. This is the reverse process to the well described technique of RBC sphering, see e.g. [32].

We use meshes of either four or five subdivisions of an icosahedron, corresponding to 642 and 2562 nodes, respectively.

### Contact mechanics of a triangulated surface

#### Local curvature of the 3D shape

Interaction between a surface and its surroundings is calculated as the interaction between two spheres, since this is an implicit requirement for Maugis-Dugdale theory. To that end, the encompassing sphere of each surface triangle is used. The outward side of the triangle is defined to be convex. This is a practical consideration: theory only requires 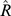 to be positive – see equation 4 – so in cases where particles with only relatively high convex curvature come in contact with particle(s) with relatively lower concave curvature (e.g. cells in a test-tube), this restriction can be relaxed. The radius of the encompassing sphere is calculated to correspond to the local inverse curvature of the triangulated surface. The inverse curvature of a triangle is calculated as the mean curvature of the three corner points, each weighted by their corresponding Voronoi region in the triangle. The curvature at each corner point 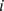 can be calculated as [33]:
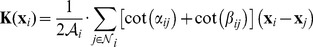
(14)

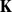
 is called the Laplace-Beltrami operator, and its 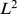-norm is twice the mean curvature while it points to the outward direction at this node. The variables in equation 14 are defined in Figure 8(c) and the sum runs over all first order neighbors of node 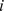, which are shown in the figure.

It should be noted, that a minimum curvature 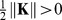 is prescribed to avoid “infinite” radii. This becomes necessary to calculate contact forces in completely flat parts of the contact – here, the contact force is generally close to zero since the contact should be already equilibrated. Although the calculation of the adhesive range 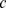 in MD theory loses accuracy by this artificial curvature, the force integration should still be a reasonable approximation, since all integration points (see below) can be expected to be in the “close contact” range 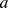 in this case.

#### Integrating the force on a triangle from the pressure distribution

When two triangulated surfaces come into contact, the contact potential is calculated from the overlap of their respective encompassing spheres. For two contacting spheres, there will be a circular contact area between the two of them, which also defines the direction of “normal” and “tangential” forces for this contact. If the two spheres are physical spheres, the contact point 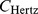 will always be located at the center of this circular area since at this point the overlap distance 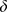 (see Figure 8(b)) will be maximal. In the case of contacting triangles, however, only a fragment of the sphere is physical and it has to be checked that a contact force needs to be calculated – supplementary Text S1 details how that can be done for any pair of rounded triangles. The cases of a contact with a sphere or a (polygonal) plane are dealt with analogously.

If the check asserts that a contact force can be expected between the triangles (or the triangle and a plane, etc.), for computational reasons we distinguish two regimes: In the first case, the contact area between the encompassing spheres is relatively large (see below, equation 18). In this case, we can assume a relatively big, well established contact between the two surfaces. Therefore, we need to integrate the pressures in equations 5 and 7. This integral is approximated using quadrature rules for numerical integration [34], [35]. For integrating any function 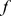 over a triangle surface 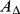, the approximation has the form:
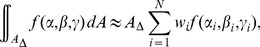
(15)in which 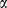, 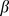 and 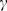 are barycentric coordinates inside the triangle, and 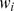 are the weights assigned to each quadrature point 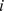.

To calculate both forces and moments caused by a specific pressure/traction of the triangle, we first determine the coordinates of the integration test points. From these points, the squared distance 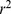 from the center of the circle of contact can be calculated. Using equation 15 we then evaluate the weighed sum, thus approximating the double integral for the force on a triangle:
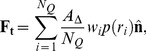
(16)where 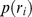 is the sum of the adhesive Maugis-Dugdale pressure (equation 7) and Hertz' repulsion (equation 5), and 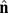 is the normal unit vector to the contact plane; 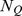 is the number of quadrature points. The divergence in the JKR adhesive stress (equation 13) makes it difficult to numerically integrate. For this reason and the added flexibility of MD theory, we chose this more general framework. Since the radius of intimate contact, 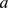, is directly known as the radius of the intersection circle of the two encompassing spheres, we only have to solve equation 11 numerically for 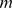 to obtain the adhesive contact radius 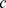 (used in equation 7).

The pressure 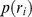 is evaluated in the positions corresponding to those quadrature points. Additionally, we sum up the moments of each individual force component with respect to the center of the contact plane:
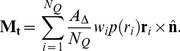
(17)


To ensure sufficient precision at an adequate speed, we use a 16-point quadrature rule of degree eight [35] that is still acceptably fast, since calculations only take place for triangles for which contact has been ascertained.

If the area of contact between the two encompassing spheres is relatively small compared to the typical area of each integration point:
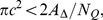
(18)we can expect a bad approximation for force and moment. Therefore, a different approach is chosen: The integrated MD force (equation 12) calculated from the total area of contact of the encompassing spheres can be scaled with the fraction of the area, which is contained in the intersection of the two triangles. This total force is then applied to the contact point 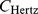, if the point is within the triangle's intersection, or the point closest to it in that intersection polygon. In this case, the moment is still calculated according to equation 17, although the sum only contains the one force and radius vector.

This second approximation for the forces and the moments one triangle of the body is subject to, is insufficient for bigger overlaps, because the moments generated by the repulsive and adhesive pressures described in equations 5 and 7 differ profoundly from that simple approximation. For small overlaps, it is obvious from equation 17 that the moment is close to 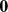 since the lever length 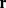 is very short, anyway.

The contact force calculated in this way does not depend on the chosen mesh – see supplementary Text S2.

#### Distribution of force to the nodes of the triangulation

To calculate the force at each node of the triangle, both the force vector and the moment vector must be taken into account. The moment-vector necessarily lies in the contact plane, since the force is defined to be normal to this plane. Let the contact plane without loss of generality be the 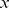-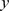 plane. This implies that 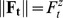 and the position vectors of the 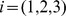 nodes w.r.t. the Hertz contact point are 
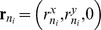
. Then, the system of equations can be conveniently written as
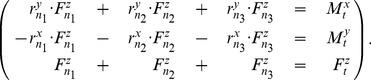
(19)This system can be inverted to find the correct forces on the nodes of the triangulation.

### Elastic model of the cortex

In the deformable cell model, the cortex nodes interact through viscoelastic potentials. In the most simple approach, a linear elastic spring could be used. For a given displacement of nodes 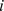 and 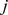, the elastic spring force over a connection is:
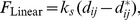
(20)in which 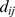 and 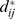 are the actual distance and equilibrium distance between connected node 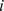 and 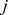. The linear spring stiffness is called 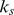. For red blood cells, two non-linear spring models have been used in literature: the finite extensible non-linear elastic model (FENE) and the worm-like chain model (WLC) [3]. These models express that upon stretching, the biopolymers of the cytoskeleton – a sub-membranous network of spectrin connections for RBCs – first uncoil, providing relatively little resistance, but when completely stretched out, become practically non-extensible.

Between two connected nodes 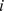 and 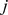, the FENE attractive potential reads:
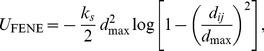
(21)where 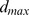 is the maximal distance, and 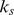 the stretching constant. The force derived from this is:
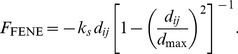
(22)


FENE springs exert purely attractive forces. In order to account for the (limited) incompressibility of the spectrin, a simple power law is used (power 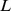):
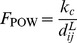
(23)The incompressibility coefficient 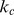 can be derived for the assumption that the total force must vanish for 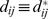, the equilibrium distance:
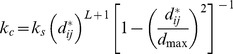
(24)In the present model, we set 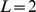, as suggested by [3]. It is convenient to denote the maximal stretch 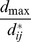 by 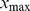, the fraction of maximal extension and equilibrium distance. In addition to this purely elastic potential, we also include dissipation as per the Kelvin-Voigt model by adding a parallel dashpot with the damping constant 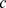:
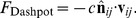
(25)Here, 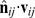 is the projection of the relative velocity of a pair of connected cortex nodes on their connecting axis. The force is also applied in the direction of the connection.

Whereas in-plane stretching and compressive forces can be calculated purely based on the distance between two neighboring cortex nodes, bending forces are calculated for two neighboring triangles. The bending moment between two adjacent triangles is given as
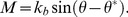
(26)Here, 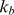 is the model parameter determining the bending rigidity, 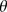 is the instantaneous angle and 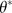 the spontaneous angle between a pair of triangles with a common edge. A corresponding force is applied to the non-common points of each of the two triangles, with a compensating force applied to the points on the common edge, ensuring that the total force on the cell remains unchanged. This type of bending-stiffness is commonly found in the literature for RBC models, eg. by [4] and [36] - a more general analysis is provided by [37].

Additionally, both a global and local area constraint is used, making sure that both the individual triangle areas and the total area of the red blood cell cannot strongly increase or decrease. As described by [4], this is achieved by a local force with magnitude:
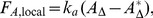
(27)in which 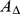 is the triangle area, 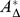 the resting triangle area and 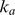 the local constraint constant. The magnitude of the global force is formulated as:
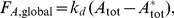
(28)where 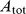 is the total RBC area, 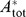 the total resting area and 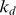 the global constraint constant. For both constants, values were taken from [3]. These forces are applied in the plane of each triangle in the direction from the barycenter of the triangle.

Finally, we add a volume constraint since for short timescales, the total cytosol volume of the cell can be considered constant. As for the area, magnitude of the force takes the form
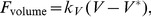
(29)with the instantaneous cell volume 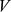 and the initial cell volume 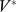. This force is applied to each node of the cell in its outward direction as found by the Laplace-Beltrami operator, see equation 14.

### Equation of motion

In the low Reynolds number environment in which cells live, motion is dominated by viscous forces [38]. In other words, inertial forces are negligible. For each integration node, Newton's second law (with explicit Stokes' drag)
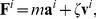
(30)by leaving out the inertial term, becomes
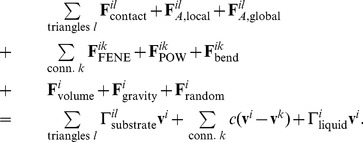
(31)The total force on node 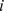 is the sum of all the individual forces: Firstly, the forces that are calculated on the triangles are transferred to the nodes – the contact forces 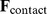 only exist for triangles, which are in contact with the substrate. Also, the local and global area constraints for the membrane are added here. Secondly, the cortex connection forces between node 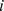 and all fixed connections 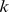 are added, and finally the volume constraint and the gravitational force 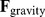 as well as a random force 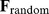 The total force on node *i* is the sum of all the individual forces: Firstly, the forces that are calculated on the triangles are transferred to the nodes – the contact forces F_contact_ only exist for triangles, which are in contact with the substrate. Also, the local and global area constraints for the membrane are added here. Secondly, the cortex connection forces between node *i* and all fixed connections *k *are added, and finally the volume constraint and the gravitational force F_contact_ as well as a random force F_contact_ for taking into account fluctuations of the membrane can be added. Since those fluctuations do not much influence the spreading dynamics in our simulations, we neglect that term for the results presented.

For the right-hand side, we not only discard the term proportional to mass, but we also more explicitly state the components of the constant 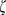: starting with the dissipative/friction term generated from the encompassing sphere - substrate friction between two contacting triangles 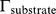. This coefficient is weighted by the distance of the node 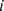 from the contact point in that triangle. This ensures symmetry of the friction-matrix (see below) and corresponds to the distribution of the contact force. The component of the substrate friction for a triangle is defined as (compare to e.g. [39]) 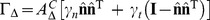 where 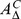 is the area of contact in that triangle, 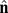 is the normalized direction vector between the two encompassing spheres and 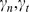 are, respectively, the normal and tangential friction constants.

Secondly, we have the dissipative dashpot of the connections of this node, and lastly we add the drag coefficient 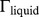 for the whole cell in plasma: here, in a first order approximation, we simply divide the formula from Stokes' law by the number of nodes per cell, thereby recapturing the exact result for a spherical cell in Stokes flow.

For nodes, whose surrounding triangles are all in contact with the substrate, we define a very high friction constant 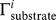, effectively fixing those nodes in place. We found that this has no influence on the spreading curves (it can be completely left out), but helps to dampen out small numerical fluctuations in the stiff potential of the contacting plane. This allows us to use larger time steps 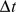 when solving this equation of motion.

Equation 31, which is used in essentially the same form by e.g. [13], [39]–[43], is a first order differential equation, which couples the movements of all particles together. When writing the whole system as
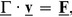
(32)it can be shown [13], that the matrix 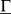 is positive definite, and therefore we are able to solve the system iteratively for the velocities by using the conjugate gradient method. Subsequently, the nodes' movement is integrated by a forward Euler scheme [44]. For a low Reynolds number environment, the amount of kinetic energy (or motion) directly corresponds to the amount of dissipated energy. Equation 32 shows all dissipative terms in the matrix 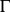 dictating the degree of motion induced by the forces 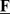. Identifying all significant dissipative mechanisms is therefore crucial for calculating the dynamics of this system.

## Supporting Information

Figure S1
**Spreading rates to assess importance of parameters.** Variation of most influential model parameters. Double-logarithmic plots of cell spreading rate (rate of contact area increase) 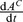 versus time. It should be noted that the numerical differentiation applied to the contact area magnifies some of the noise which is due to discretization in the spreading rate. (**a**) varying cell-substrate adhesion strength 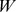 yields both a shift in the initial rate and final contact radius. (**b**) varying the FENE stretching constant 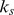 yields different final contact radii, but the rates are comparable, (**c**) varying the FENE max stretch 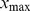 also mostly influences the final contact radii, (**d**) varying bending stiffness 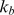 influences both spreading rate and final contact radius, (**e**) varying the normal friction coefficient 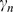 influences spreading rate and (**f**) varying the local area constraint constant 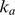 influences the final spreading rates.(EPS)

Figure S2
**Cortex stretch during RBC spreading.** Stretch (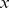 [-]) in the FENE connections of the RBC membrane averaged at the nodes at different time points during cell spreading. left: 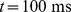, middle: 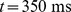, right: 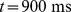.(TIF)

Text S1 **Resolution of contact and calculation of the contact point for two rounded triangles.**
(PDF)

Text S2 **Bouncing ball simulation and mesh independence of the contact force.**
(PDF)

Video S1 **Simulated spreading of typical biconcave-shaped RBC on adhesive substrate.** Arrows show the magnitude and direction of the sum of conservative force in the nodes, color represents the magnitude of the sum of all conservative forces. The movie is slowed down – the total spreading time is less than a second.
(MPG)

Video S2 **Simulated spreading of rounded RBC on adhesive substrate.** Arrows show the magnitude and direction of the sum of conservative force in the nodes, color represents the magnitude of the sum of all conservative forces. The movie is slowed down – the total spreading time is less than a second.
(MPG)

Video S3 **Comparison of different meshes of bouncing ball.** In one simulation, we use the exact Hertz-solution of a bouncing-ball simulation (left, perfect sphere), as well as three refinements of the triangulated model explained in the text. Small differences in bouncing height can be seen at later times for the coarsest mesh.
(MPG)

## References

[1] Cuvelier D, Thery M, Chu YS, Dufour S, Thiery JP, et al (2007) The universal dynamics of cell spreading. Current Biology 17: 694–699. 17379524 10.1016/j.cub.2007.02.058

[2] Huveneers S, Danen EHJ (2009) Adhesion signaling C crosstalk between integrins, src and rho. Journal of Cell Science 122: 1059–1069. 19339545 10.1242/jcs.039446

[3] Fedosov DA, Caswell B, Karniadakis GE (2010) Systematic coarse-graining of spectrin-level red blood cell models. Computer Methods in Applied Mechanics and Engineering 199: 1937–1948. 10.1016/j.cma.2010.02.001 PMC3864857 24353352

[4] Fedosov DA, Lei H, Caswell B, Suresh S, Karniadakis GE (2011) Multiscale modeling of red blood cell mechanics and blood flow in malaria. PLoS Computational Biology 7: e1002270. 22144878 10.1371/journal.pcbi.1002270 PMC3228770

[5] Lu M, McDowell G (2007) The importance of modelling ballast particle shape in the discrete element method. Granular Matter 9: 69–80.

[6] Matuttis H, Luding S, Herrman H (2000) Discrete element simulations of dense packings and heaps made of spherical and non-spherical particles. Powder Technology 108: 278–292.

[7] Pöschel T, Schwager T (2005) Computational Granular Dynamics. Springer.

[8] Spillmann J, Teschner M (2005) Contact surface computation for coarsely sampled deformable objects. In: Proc. Vision, Modeling, Visualization.

[9] Drasdo D, Hoehme S, Block M (2007) On the role of physics in the growth and pattern formation of multi-cellular systems: What can we learn from individual-cell based models. Journal of Statistical Physics 128: 287–345.

[10] Dao M, Li J, Suresh S (2006) Molecularly based analysis of deformation of spectrin network and human erythrocyte. Materials Science and Engineering: C 26: 1232–1244.

[11] Hategan A, Sengupta K, Kahn S, Sackmann E, Discher DE (2004) Topographical pattern dynamics in passive adhesion of cell membranes. Biophysical Journal 87: 3547–3560. 15339814 10.1529/biophysj.104.041475 PMC1304820

[12] Suresh S, Spatz J, Mills J, Micoulet A, Dao M, et al (2005) Connections between single-cell biomechanics and human disease states: gastrointestinal cancer and malaria. Acta Biomaterialia 1: 15–30. 16701777 10.1016/j.actbio.2004.09.001

[13] Van Liedekerke P, Smeets B, Odenthal T, Tijskens E, Ramon H (2013) Solving microscopic flow problems using stokes equations in sph. Computer Physics Communications 184: 1686–1696.

[14] Leckband D, Israelachvili J (2001) Intermolecular forces in biology. Quarterly Reviews of Biophysics 34 ((2)) 105–267. 11771120 10.1017/s0033583501003687

[15] Hategan A, Law R, Kahn S, Discher DE (2003) Adhesively-tensed cell membranes: Lysis kinetics and atomic force microscopy probing. Biophysical Journal 85: 2746–2759. 14507737 10.1016/s0006-3495(03)74697-9 PMC1303498

[16] Seifert U, Lipowsky R (1990) Adhesion of vesicles. Physical Review A 42 ((8)) 4768–4771. 10.1103/physreva.42.4768 9904586

[17] Boal D (2012) Mechanics of the Cell. Cambridge University Press, 2 edition.

[18] Wang N, Ostuni E, Whitesides G, Ingber D (2002) Micropatterning tractional forces in living cells. Cell Motility and the Cytoskeleton 52: 97–106. 12112152 10.1002/cm.10037

[19] Legant W, Choi C, Miller J, Shao L, Gao L, et al (2013) Multidimensional traction force microscopy reveals out-of-plane rotational moments about focal adhesions. PNAS 110 ((3)) 881–886. 23277584 10.1073/pnas.1207997110 PMC3549134

[20] Brady J, Bossis G (1988) Stokesian dynamics. Anuual Review of Fluid Mechanics 20: 111–157.

[21] Israelachvili JN (2011) Intermolecular and surface forces. Academic press, third edition.

[22] Xiong Y, Rangamani P, Fardin MA, Lipshtat A, Dubin-Thaler B, et al (2010) Mechanisms controlling cell size and shape during isotropic cell spreading. Biophysical journal 98: 2136–2146. 20483321 10.1016/j.bpj.2010.01.059 PMC2872297

[23] Kim MC, Neal DM, Kamm RD, Asada HH (2013) Dynamic modeling of cell migration and spreading behaviors on fibronectin coated planar substrates and micropatterned geometries. PLOS Computational Biology 9: e1002926. 23468612 10.1371/journal.pcbi.1002926 PMC3585413

[24] Maugis D (1992) Adhesion of spheres: The jkr-dmt transition using a dugdale model. Journal of Colloid and Interface Science 150: 243–269.

[25] Johnson KL, Greenwood JA (1997) An adhesion map for the contact of elastic spheres. Journal of colloid and interface science 192: 326–333. 9367554 10.1006/jcis.1997.4984

[26] Johnson K (1997) Adhesion and friction between a smooth elastic spherical asperity and a plane surface. Proceedings of the Royal Society of London Series A: Mathematical, Physical and Engineering Sciences 453: 163–179.

[27] Johnson KL, Kendall K, Roberts A (1971) Surface energy and the contact of elastic solids. Proceedings of the royal society 324: 301–313.

[28] Barthel E (2008) Adhesive elastic contacts: Jkr and more. Journal of Physics D: Applied Physics 41: 163001.

[29] Chu YS, Dufour S, Thiery JP, Perez E, Pincet F (2005) Johnson-kendall-roberts theory applied to living cells. Physical Review Letters 94: 028102. 15698233 10.1103/PhysRevLett.94.028102

[30] Van Liedekerke P, Tijskens E, Ramon H, Ghysels P, Samaey G, et al (2010) Particle-based model to simulate the micromechanics of biological cells. PHYSICAL REVIEW E 81: 061906-1–061906-15. 10.1103/PhysRevE.81.061906 20866439

[31] Xu G (2006) Discrete laplace–beltrami operator on sphere and optimal spherical triangulations. International Journal of Computational Geometry & Applications 16: 75–93.

[32] Fung Y, Tong P (1968) Theory of the sphering of red blood cells. Biophysical journal 8: 175–198. 5639934 10.1016/S0006-3495(68)86484-7 PMC1367371

[33] Meyer M, Desbrun M, Schröder P, Barr AH (2002) Discrete differential-geometry operators for triangulated manifolds. Visualization and Mathematics 3: 35–57.

[34] Cowper GR (1973) Gaussian quadrature formulas for triangles. International Journal for Numerical Methods in Engineering 7: 405–408.

[35] Zhang L, Cui T, Liu H (2009) A set of symmetric quadrature rules on triangles and tetrahedra. J Comput Math 27: 89–96.

[36] Discher DE, Boal DH, Boey SK (1998) Simulations of the erythrocyte cytoskeleton at large deformation. ii. micropipette aspiration. Biophysical Journal 75: 1584–1597. 9726959 10.1016/S0006-3495(98)74076-7 PMC1299832

[37] Boal DH, Rao M (1992) Topology changes in fluid membranes. Physical Review A 46: 3037–3045. 10.1103/physreva.46.3037 9908473

[38] Purcell E (1977) Life at low reynolds-number. American Journal of Physics 45: 3–11.

[39] Hoehme S, Drasdo D (2005) A single-cell-based model of tumor growth in vitro: monolayers and spheroids. Physical Biology 2: 133–147. 16224119 10.1088/1478-3975/2/3/001

[40] Galle J, Loeffler M, Drasdo D (2005) Modeling the effect of deregulated proliferation and apoptosis on the growth dynamics of epithelial cell populations in vitro. Biophysical journal 88: 62–75. 15475585 10.1529/biophysj.104.041459 PMC1305039

[41] Ramis-Conde I, Chaplain MAJ, Anderson ARA, Drasdo D (2009) Multi-scale modelling of cancer cell intravasation: the role of cadherins in metastasis. Physical Biology 6: 016008. 19321920 10.1088/1478-3975/6/1/016008

[42] Krinner A, Hoffmann M, Loeffler M, Drasdo D, Galle J (2010) Individual fates of mesenchymal stem cells in vitro. BMC Systems Biology 4: 1–9. 20507570 10.1186/1752-0509-4-73 PMC2901224

[43] Hoehme S, Brulport M, Bauer A, Bedawy E, Schormann W, et al (2010) Prediction and validation of cell alignment along microvessels as order principle to restore tissue architecture in liver regeneration. Proceedings of the National Academy of Sciences 107: 10371–10376. 10.1073/pnas.0909374107 PMC2890786 20484673

[44] Tijskens E, Ramon H, Baerdemaeker JD (2003) Discrete element modelling for process simulation in agriculture. Journal of Sound and Vibration 266: 493–514.

