# Supplementary figures and images for: Analysis of Initial Cell Spreading Using Mechanistic Contact Formulations for a Deformable Cell Model
Source: PLoS Comput Biol. 2013 Oct 17;9(10):e1003267. doi: 10.1371/journal.pcbi.1003267 (PMC3798278; doi:10.1371/journal.pcbi.1003267)

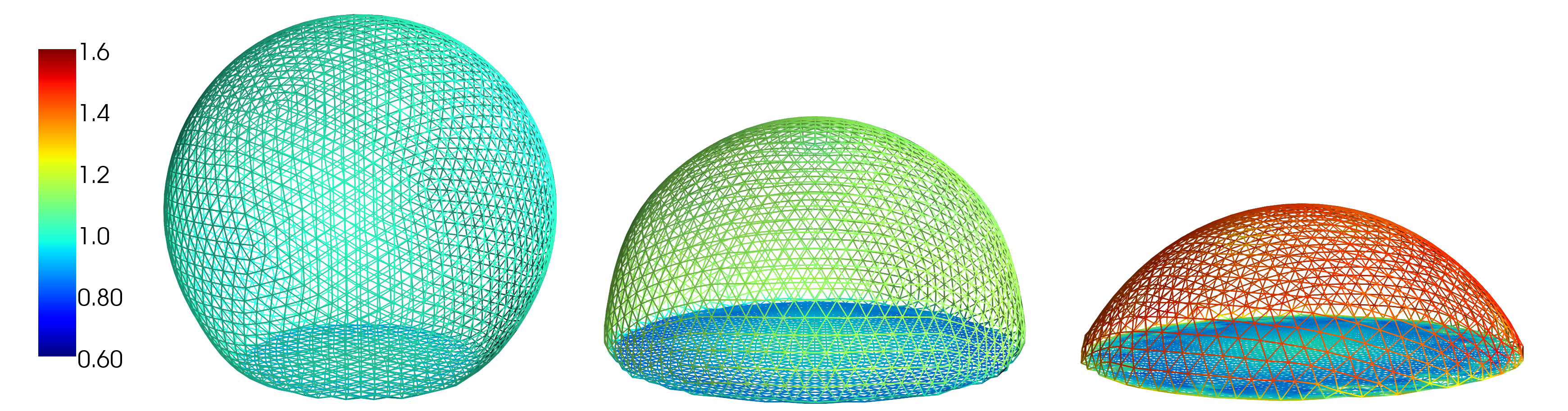

Supplement: Figure S2 — Cortex stretch during RBC spreading. Stretch ( [-]) in the FENE connections of the RBC membrane averaged at the nodes at different time points during cell spreading. left: , middle: , right: . (TIF) [file pcbi.1003267.s002.tif]
