# Supplementary material for: Analysis of Initial Cell Spreading Using Mechanistic Contact Formulations for a Deformable Cell Model
Source: PLoS Comput Biol. 2013 Oct 17;9(10):e1003267. doi: 10.1371/journal.pcbi.1003267 (PMC3798278; doi:10.1371/journal.pcbi.1003267)
Supplement: Text S1 — Resolution of contact and calculation of the contact point for two rounded triangles. (PDF) [file pcbi.1003267.s003.pdf]

Supplementary Information 1:
Resolution of contact and contact point for two rounded triangles

Here we derive the contact point between two contacting triangles, which have encompassing spheres. We assume that the actual contact point must be within the contact plane of the spheres. Any other position of the contact point would "favor" one sphere over the other for no apparent reason. Therefore, we first orthogonally project the corner points of both triangles $P = \{p_1, p_2, p_3\}$ and $Q = \{q_1, q_2, q_3\}$ to the contact plane of the spheres, as illustrated in Figure S1.

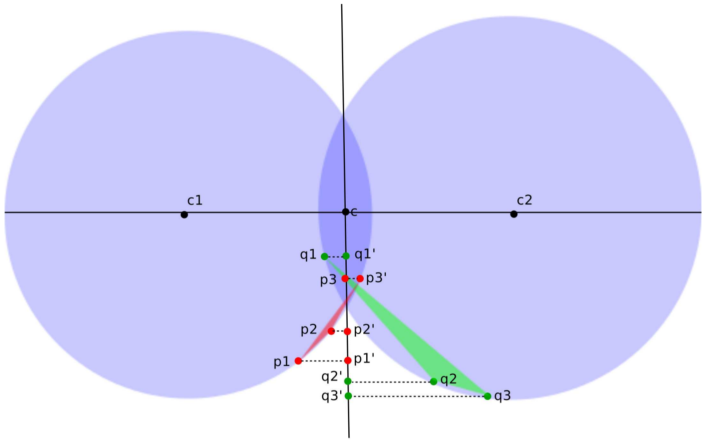

Figure S1. Triangles P and Q, which belong to two (deformable) bodies in contact, are projected onto the contact plane of their encompassing spheres. This is needed to check, if a contact force and a contact moment is generated from this particular pair of facets, and if so to calculate the magnitudes.

The overlap area and the two projected triangles $P' = \{p_{1'}, p_{2'}, p_{3'}\}$ and $Q' = \{q_{1'}, q_{2'}, q_{3'}\}$ are all located in the same plane, which is shown in Figure S2. An actual contact between the triangles exists when there exists an intersecting area of both contact circle, P' and Q' (indicated by the dark red area in Figure S2). As the overlap between the encompassing spheres decreases monotonously when going away from c, the contact point where the overlap between the encompassing spheres is maximal must be the point in the dark red area that is closest to c. This point is also the point in the light red area that is closest to c, as the extra part in the light red area is outside the circle and therefore always further away from c.

The light red area is the polygon $S = \{q_{1'}, s_1, p_{3'}, s_2\}$. The number of corner points of this polygon can vary dependent on the relative positions of the two triangles, and can take values between 3 and 6. In order to fully determine this polygon we need to calculate the intersection points (in this case s_1 and s_2). The intersection s between two lines defined by four points x_1, x_2, x_3 and x_4 can be found as:

$$s = x_1 + a\frac{(c \times b) \cdot (a \times b)}{\|a \times b\|^2} \tag{33}$$

with $a = x_2 - x_1$, $b = x_4 - x_3$ and $c = x_3 - x_1$. Of course we will always find an intersection between two lines within a plane. We also need to check whether s is effectively on the triangle segment. For a triangle segment between p_1 and p_2, this is true if:

$$\|p_1 - s\|^2 < \|p_1 - p_2\|^2 \tag{34}$$

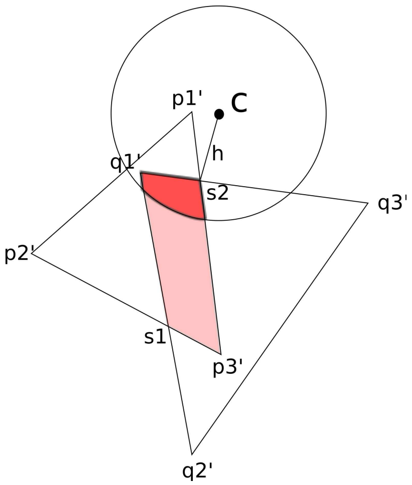

Figure S2. Intersection of triangles $P' = \{p_{1'}, p_{2'}, p_{3'}\}$, $Q' = \{q_{1'}, q_{2'}, q_{3'}\}$ and the contact area of the two encompassing spheres of the triangles with center c.

and

$$\|p_2 - s\|^2 < \|p_1 - p_2\|^2 . \tag{35}$$

This check requires no (expensive) calculations of square roots. In order to find all intersection points between two triangles P' and Q' we have to calculate equation 33 nine times (three times for each side of P') and perform 18 checks of the squared norms.

The polygon S is the collection of all intersection points and all corner points of the triangles P' that are within triangle Q' and corner points of triangle Q' that are within triangle P'. This requires six checks again. Next, we sort each point p_i from $\{p_2 \dots p_n\}$ according to the dot product with p_1, yielding a set of counter-clockwise sorted elements. We then calculate the closest distance from this sorted polygon S to c, giving the closest point c_p as the contact point and the squared distance h^2. h^2 can be used to calculate the overlap δ_t. If c is within S, the distance between S and c is zero and the contact point will be c itself. From h^2, we can calculate the actual overlap δ_t as:

$$\delta_t = \delta_s - R_1 - R_2 + \sqrt{R_1^2 - h^2} + \sqrt{R_2^2 - h^2} \tag{36}$$

with $h = \|c - c_p\|^2$ and δ_s being the overlap for a sphere-sphere contact ($\delta_s = \|c_2 - c_1\| - R_1 - R_2$).

An additional check that we need to perform for this method is whether one of the two triangles actually is on the opposite side of the sphere. We only need to calculate contacts between triangles which are facing each other with their "outside" direction. We define the threshold angle for this to be $90\,^\circ$, i.e. for angles of more than $90\,^\circ$ between any triangle and the contact plane, the triangles do not face each other and no contact should be calculated:

$$n_1 \cdot (c_2 - c_1) > 0 \quad \wedge \quad n_2 \cdot (c_1 - c_2) > 0 \tag{37}$$

in which n_1 and n_2 are normal vectors on triangle 1 (P) and triangle 2 (Q). If equation 37 holds, the triangles are facing each other. In the implementation, this check is performed at the start of contact resolution, because we can immediately discard the contact if this simple check does not hold.
