# Supplementary material for: Analysis of Initial Cell Spreading Using Mechanistic Contact Formulations for a Deformable Cell Model
Source: PLoS Comput Biol. 2013 Oct 17;9(10):e1003267. doi: 10.1371/journal.pcbi.1003267 (PMC3798278; doi:10.1371/journal.pcbi.1003267)
Supplement: Text S2 — Bouncing ball simulation and mesh independence of the contact force. (PDF) [file pcbi.1003267.s004.pdf]

Supplementary Information 2: Bouncing Ball and mesh independence of contact force

As a proof-of-principle problem, we let a soft, but rigid (with only six degrees of freedom) ball bounce on a soft flat substrate. The only acting forces are gravity and a velocity dependent drag force as body forces on the ball, and Hertz' contact force between the ball and the substrate. We solve Newton's equations of motion:

$$\mathbf{F}_{\text{ball}} = m \cdot \mathbf{g} - c \cdot \mathbf{v} + \frac{4\hat{E}a^3}{3\hat{R}} \cdot \hat{n} = m \cdot \mathbf{a}, \tag{38}$$

using an explicit leap-frog time integration method [44]. Here $\hat{n}$ is the normal vector to the contact plane. Figure S3 shows the result of that simulation with the rigid sphere, but also with three differently coarse meshes, using the algorithm explained in the previous sections.

Meshed bouncing ball

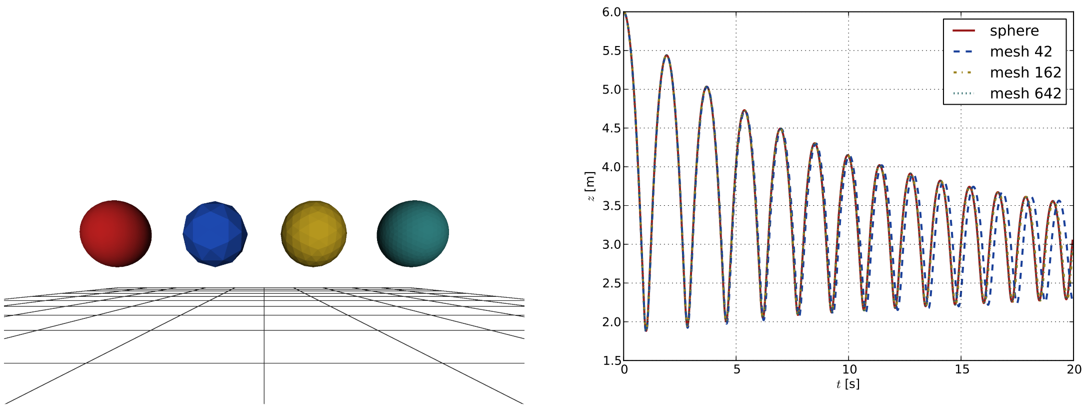

(a) (b)

Figure S3. Bouncing ball simulation. (a) gives a view on the starting point of the simulation. (b) shows the time evolution of the z-coordinate of the center of mass for all four representations of the ball. For the coarsest mesh with 42 nodes, deviations at the end of the simulation are visible. See also supplementary Video S3.

As Figure S3 (and supplementary Video S3) illustrates, the newly developed contact model achieves the same results for this situation as the well-known Hertz' model (red sphere). On top of that, the outcome of the simulations does not depend on the refinement of the chosen mesh: Only minor deviations can be seen after approximately ten "bounces" for the coarsest mesh.

Meshed sphere adhesion

We show convergence with mesh refinement by simulating an adhesive triangulated sphere ($R = 3\mu$m, $W = 1$mJ/m^2) that is being loaded onto a flat plane. Five different mesh refinements are considered, all based on regular subdivisions of the icosahedron yielding 48 to 20 242 nodes. Figure S4(a) shows force versus penetration depth for subdivisions with 48, 642 and 10 242 nodes, calculated by integrating the MD pressure for a Tabor coefficient (μ) of 0.1, compared to the solution of Hertz (no adhesion), JKR, MD

and DMT. Figure S4(b) shows the change in normalized Root Mean Square Error (RMSE) with mesh refinement for different Tabor coefficents μ. For high values of μ (close to the JKR limit), the error in the calculated forces is larger as the adhesive tension diverges near the edge of the contact radius (compare to equations 7 and 13).

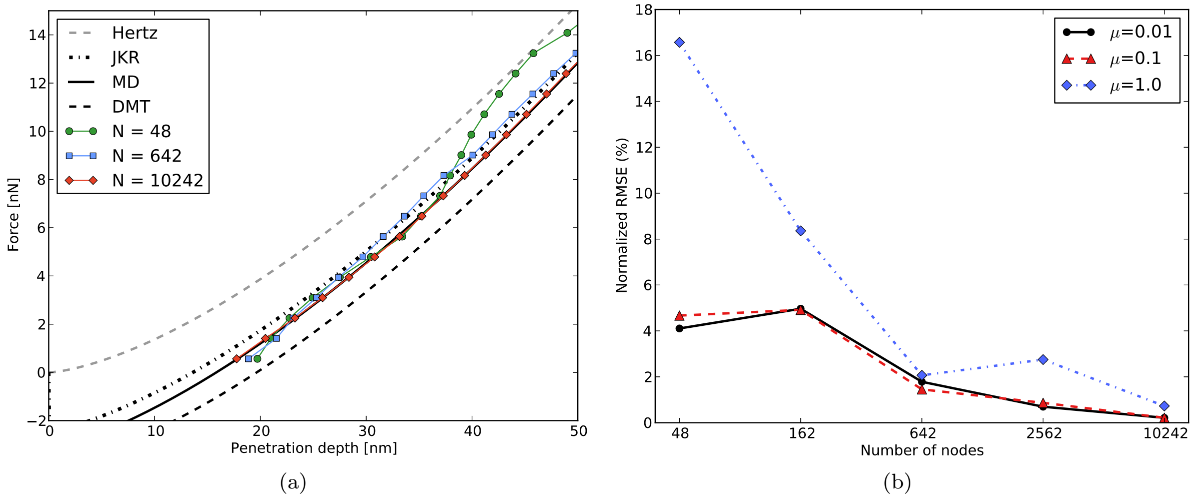

Figure S4. Adhesive sphere simulation. (a): Force versus penetration depth for adhesive meshed sphere (MD, $\mu = 0.1$) with different mesh refinements, compared with analytical solutions for Hertz, JKR, MD and DMT. (b): Root Mean Square Error (RMSE), normalized as a percentage of the maximal force, for prediction of the analytical MD solution as a function of mesh refinement for different Tabor coefficients μ.
